# Genome-wide patterns of copy number variation in the diversified chicken genomes using next-generation sequencing

**DOI:** 10.1186/1471-2164-15-962

**Published:** 2014-11-07

**Authors:** Guoqiang Yi, Lujiang Qu, Jianfeng Liu, Yiyuan Yan, Guiyun Xu, Ning Yang

**Affiliations:** Department of Animal Genetics and Breeding, College of Animal Science and Technology, China Agricultural University, Beijing, China

**Keywords:** Copy number variation, Whole genome sequencing, aCGH, Genetic diversity, Chicken

## Abstract

**Background:**

Copy number variation (CNV) is important and widespread in the genome, and is a major cause of disease and phenotypic diversity. Herein, we performed a genome-wide CNV analysis in 12 diversified chicken genomes based on whole genome sequencing.

**Results:**

A total of 8,840 CNV regions (CNVRs) covering 98.2 Mb and representing 9.4% of the chicken genome were identified, ranging in size from 1.1 to 268.8 kb with an average of 11.1 kb. Sequencing-based predictions were confirmed at a high validation rate by two independent approaches, including array comparative genomic hybridization (aCGH) and quantitative PCR (qPCR). The Pearson’s correlation coefficients between sequencing and aCGH results ranged from 0.435 to 0.755, and qPCR experiments revealed a positive validation rate of 91.71% and a false negative rate of 22.43%. In total, 2,214 (25.0%) predicted CNVRs span 2,216 (36.4%) RefSeq genes associated with specific biological functions. Besides two previously reported copy number variable genes *EDN3* and *PRLR*, we also found some promising genes with potential in phenotypic variation. Two genes, *FZD6* and *LIMS1*, related to disease susceptibility/resistance are covered by CNVRs. The highly duplicated *SOCS2* may lead to higher bone mineral density. Entire or partial duplication of some genes like *POPDC3* may have great economic importance in poultry breeding.

**Conclusions:**

Our results based on extensive genetic diversity provide a more refined chicken CNV map and genome-wide gene copy number estimates, and warrant future CNV association studies for important traits in chickens.

**Electronic supplementary material:**

The online version of this article (doi:10.1186/1471-2164-15-962) contains supplementary material, which is available to authorized users.

## Background

Copy number variations (CNVs) are defined as gains or losses of DNA fragments of 50 bp or longer in length in comparison with reference genome [1, 2]. CNVs contribute significantly to both disease susceptibility/resistance and normal phenotypic variability in humans [3–5] and animals [6–9]. Four major mechanisms have been found to be related to CNV formation including non-allelic homologous recombination (NAHR), non-homologous end joining (NHEJ), Fork Stalling and Template Switching (FoSTeS) and LINE1 Retrotransposition [4, 10]. Additionally, segmental duplications (SDs) which are duplicated sequences (insertions) of ≥1 kb in length and ≥90% sequence identity are also suggested to be one of the major catalysts and hotspots for CNV formation [11, 12], mainly because the genomic regions flanked by SDs are susceptible to recurrent rearrangement by NAHR [11, 13]. In terms of total bases involved, the percentage of the genome affected by CNVs is higher than that of single nucleotide polymorphism (SNP) markers. Although SNPs are generally considered as more suitable markers in the genome-wide association studies (GWASs), most reported SNP variants have relatively limited effects and explain only a small proportion of phenotypic variance [14]. Further, CNVs encompassing genes or regulatory elements are believed to exert potentially larger effects on gene expression through changing gene structure and dosage, altering gene regulation, exposing recessive alleles and other mechanisms [1, 4, 15, 16]. CNVs are also found to alone capture 18 to 30% of the total detected genetic variation in gene expression in humans and animals, and may contribute to a fraction of the missing heritability [17, 18]. Therefore, identification of CNVs is essential in whole genome fine-mapping of CNVs and association studies for important phenotypes.

Originally, two cost-effective and high-throughput methods including array comparative genomic hybridization (aCGH) and commercial SNP microarrays are used for CNV screening [19, 20]. However, due to the limitation in resolution and sensitivity, it is difficult with the two approaches to detect small CNVs shorter than 10 kb in length and identify the precise boundaries of CNVs [21, 22]. The two analytical platforms also reveal inconsistent results with poor overlaps owing to different designs and probe densities and coordinates [18, 20]. Furthermore, the presence of SD regions is also a common challenge for microarrays, because a considerable proportion of CNVs fall into SD regions not well-covered by microarrays [2, 23]. Recently, a variety of CNV detection approaches based on next-generation sequencing (NGS) were proposed and offer promising alternatives as they have higher effective resolution to discover CNVs with more types and wider size ranges [24]. One leading method is read depth (RD) (also known as depth of coverage (DOC)) with the capability of inferring gains or losses of DNA segments and determining absolute copy number values, which detects CNVs by analyzing the number of reads that fall into each pre-specified window with a certain size [25, 26]. Hence, the advent of NGS technologies and suitable analytical methods can promote to systematically identify CNVs at higher resolution and sensitivity.

At present, the three aforementioned high-throughput platforms have been applied to livestock genomics for CNV detection, such as sheep [27], horse [28] and cattle [2], and uncover several CNVs associated with important phenotypes. Some CNVs are also found to be the genetic foundation of phenotypic variation in chickens. A duplicated sequence close to the first intron of *SOX5* is associated with the chicken pea-comb phenotype [29] and an inverted duplication containing *EDN3* causes dermal hyperpigmentation [30]. Partial duplication of the *PRLR* is related to the late feathering [31].

A genome-wide chicken CNV analysis is desired since chicken is not only an economically important farm animal but also a valuable biomedical model [9, 32]. However, some previous CNV studies in chickens based on aCGH and SNP platforms mainly suffered from low resolution and sensitivity [9, 32–35]. A latest study exhibited the detection of four main types of genetic variation from whole genome sequencing data using two chickens [36], suggesting the efficiency of CNV detection via deep sequencing. Considering that a great number of CNVs appears to be segregating in distinct breeds, we selected 12 chickens from multiple breeds with extensive genetic diversity, including seven Chinese indigenous breeds [37], four commercial breeds and one Red Jungle Fowl. Then we applied NGS-based method to construct a more refined and individualized chicken CNV map, investigate genome-wide CNV characteristics and estimate genome-wide gene copy number. The results will enable us to better understand the patterns of CNVs in the chicken genome and future CNV association studies.

## Results

### Mapping statistics and CNV detection

We performed whole genome sequencing in 12 different breeds of female chickens using Illumina paired-end libraries and obtained a total of 12.9 Gb high quality sequence data per individual after quality filtering. After sequence alignment and removing potential PCR duplicates, the sequencing depth varied from 8.2× (CS) to 12.4× (WR), which was sufficient for CNV detection, and the average coverage with respect to the chicken reference genome sequence was 97.2% (Table 1). We calculated the average read depth (RD) of 5 kb non-overlapping windows for all autosomes and performed GC correction. The GC-adjusted RD mean and standard deviation (STDEV) for each individual are listed in Table 1. We applied the program CNVnator to 12 individuals and the average number of CNVs per individual was 1,328, ranging from 644 in WL to 1,921 CNVs in BY. A detailed description of CNV calls can be found in Additional file 1: Table S1. For all the autosomal CNVs classified as duplications, the average copy number value of all the individuals was 3.78 and the maximum copy number estimate was 40.8 on chromosome 2 (chr2) in RJF.

**Table 1:** **Summary statistics for sequencing and CNVs of 12 individuals**

Chicken abbreviation ^a^	Numbers of mapped reads	Depth	Coverage (%)	Autosome reads per 5 kb window ^b^	Autosome reads STDEV	Duplications	Deletions	Sequence covered (Mb)
BY	102,002,937	9.7	97.0	489.29	110.73	1,319	602	34.1
CS	85,383,494	8.2	96.9	409.93	101.42	1,132	663	26.8
DX	129,847,015	12.4	97.4	623.50	130.46	552	820	8.2
LX	105,152,881	10.0	97.3	503.82	112.74	898	821	11.7
RIR	102,464,756	9.8	97.3	490.96	108.21	578	669	8.3
RJF	105,517,587	10.1	97.2	504.23	113.52	702	620	9.8
SG	85,987,827	8.2	96.6	412.27	87.66	470	553	7.2
SK	95,322,371	9.1	97.1	457.21	100.61	773	657	12.3
TB	107,535,104	10.3	97.3	515.68	108.07	607	679	8.5
WC	119,116,969	11.4	97.4	572.35	121.39	710	768	9.8
WL	118,689,980	11.3	97.5	567.18	118.63	203	441	3.3
WR	130,307,416	12.4	97.6	625.01	132.32	224	477	3.3

^a^ *BY* Beijing You, *CS* Cornish, *DX* Dongxiang, *LX* Luxi Game, *RIR* Red Island Rhode, *RJF* Red Jungle Fowl, *SG* Shouguang, *SK* Silkie, *TB* Tibetan, *WC* Wenchang, *WL* White Leghorn, *WR* White Plymouth Rock.

^b^The number of reads per 5 kb windows after GC correction.

**Figure 1:**
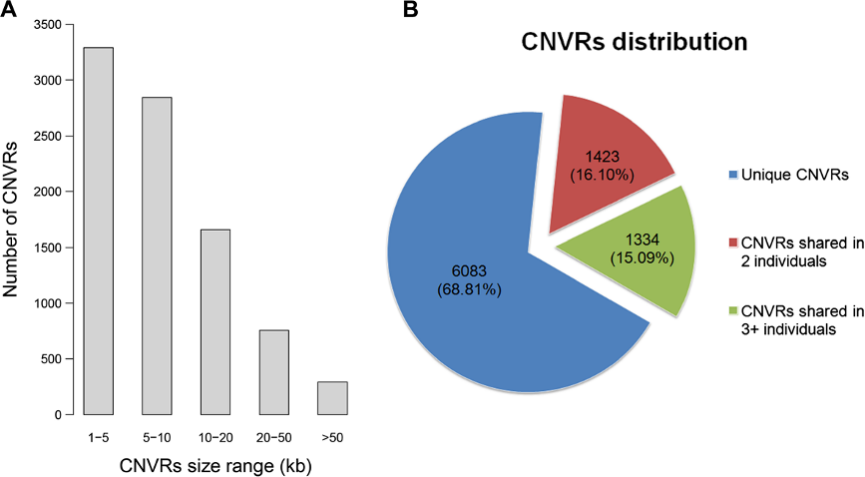
**The length and frequency distribution of CNVRs. (A)** 6,137 (69.4%) CNVR events are shorter than 10 kb, and the number of CNVRs longer than 50 kb is only 291 (3.3%). **(B)** 6,083 (68.8%) CNVR occur in only one individual and 2,757 (31.2%) CNVRs are shared in at least two individuals.

A total of 8,840 CNV regions (CNVRs) allowing for CNV overlaps of 1 bp or greater were obtained, covering chromosomes 1–28, two linkage groups and sex chromosomes, which amounted to 98.2 Mb of the chicken genome and corresponded to 9.4% of the genome sequence (Additional file 1: Table S1). The individualized chicken CNV map across the genome is shown in Additional file 2: Figure S1. The length of CNVRs ranged from 1.1 to 268.8 kb with an average of 11.1 kb and a median of 6.6 kb. In total, 6,137 (69.4%) out of all CNVRs had sizes varying from 1.1 to 10 kb (Figure 1A). Although chr1 had a maximum of 1,928 CNVRs, the two largest CNVR density values, defined as the average distance between CNVRs, were 35.7 kb and 32.0 kb on the chr16 and chrLGE64, respectively (Additional file 3: Table S2). The number of CNVRs in different individuals varied greatly, ranging from 629 in WL to 1,890 in BY. Among all CNVRs, 6,083 (68.8%) were present in a single individual, 1,423 (16.1%) shared in two individuals, and 1,334 (15.1%) shared in at least three individuals (Figure 1B). Further, the mean and median lengths of the unique CNVRs were 8.9 kb and 5.8 kb, whereas the shared CNVRs sizes were 15.9 kb on average and 9.5 kb as the median. According to the type of CNVRs, they were divided into three categories, including 4,761 gain, 3,773 loss and 306 both (gain and loss) events. Gain events possessed longer genomic sequences than losses both on average (14.2 kb vs. 5.4 kb) and in total (67.6 Mb vs. 20.3 Mb). In addition, the count of CNVRs on each chromosome was directly proportional to the chromosome length, and five macrochromosomes (chr1-5) possessed a large proportion (61.8%) of all putative CNVRs.

### Comparison with previous chicken CNV studies

Considering that most of the previous studies excluded the CNVRs on sex chromosomes and unassigned linkage groups, we migrated our autosomal CNVR coordinates from galGal4 to galGal3 using the UCSC liftOver tool. In total, 7,530 out of 8,487 (88.7%) autosomal CNVRs were converted successfully. The detailed comparison results are presented in Table 2 and Additional file 4: Table S3. In our results, 1,052 (14.0%) CNVRs with the total length of 19.7 Mb were reported by eight previous studies, and the remaining 6,478 (86.0%) were regarded as novel CNVRs. It should be noted that the novel CNVRs had slightly smaller sizes (10.6 kb) on average than those reported CNVRs (18.8 kb). As a special and important chromosome in the chicken genome, chr16 encompassed some CNVRs which could be confirmed by different platforms.

**Table 2:** **Comparison between autosomal CNVRs identified in this study and other chicken studies**

Platforms	Results from different studies					Overlapped CNVRs in this study			
	Study	Breed	Samples	Number	Total length (Mb)	Number	Pct. of number (%)	Total length (Mb)	Pct. of length (%)
Sequencing-based study	This study	12	12	7,530	88.12				
CGH-based studies	Wang et al. 2010 [40]	3	10	91	15.72	162	2.15	2.66	3.02
	Wang et al. 2012 [9]	3	6	130	3.34	83	1.10	0.92	1.04
	Crooijmans et al. 2013 [34]	7	64	1,504	57.44	721	9.58	8.30	9.42
	Luo et al. 2013 [39]	4	6	29	1.46	21	0.28	0.42	0.47
	Tian et al. 2013 [33]	11	22	308	10.81	166	2.20	2.00	2.27
	Abernathy et al. 2014 [41]	2	12	147	4.18	68	0.90	0.63	0.71
SNP-based study (60 K)	Jia et al. 2013 [32]	2	746	209	13.55	141	1.87	1.75	1.99
Sequencing-based study	Fan et al. 2013 [36]	2	2	415	3.17	96	1.27	0.80	0.90
Total						1,052	13.97	12.45	14.13

### CNV quality assessment by CNVnator, aCGH and qPCR

**Figure 2:**
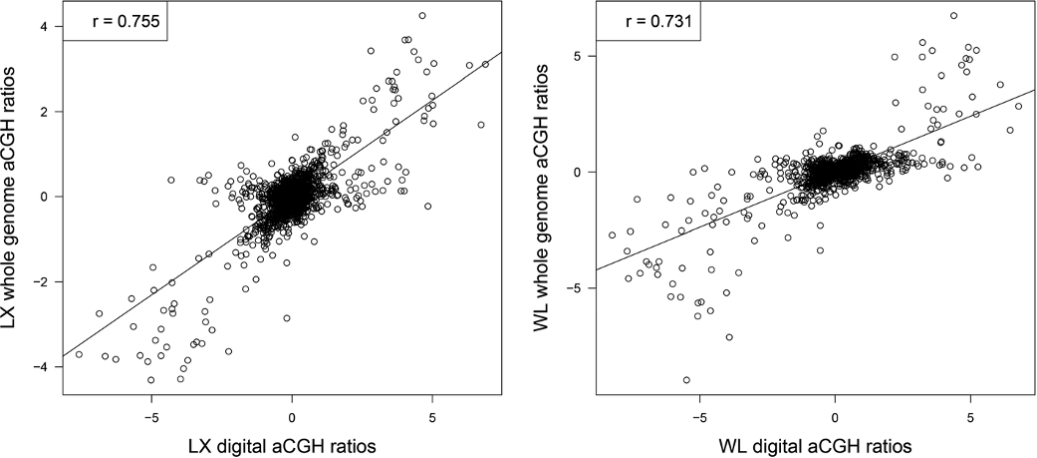
**Correlation between digital aCGH and whole genome aCGH among Luxi Game and White Leghorn compared with Red Jungle Fowl (RJF).** RJF is selected as the reference sample in each aCGH experiment. Digital aCGH values are estimated using calculated log_2_ CN ratios in which CN are estimated for identified CNV segments of two individuals and divided by the corresponding CN of RJF. Whole genome aCGH values are defined as the average of all probes log_2_ ratio values in the same segments as the digital aCGH.

The copy number values of diploid regions on autosomes theoretically equal to two, so we could inspect the potential of CNVnator to generate false positive results by evaluating these two copies regions. For all 12 individuals, we selected all 5 kb non-overlapping windows on autosomes and excluded the windows intersecting with predicted CNVs and gaps, and then estimated their average CN. The average CN and STDEV per individual was 2.077 ± 0.291, varied from 2.041 ± 0.226 in WR to 2.104 ± 0.299 in RJF, showing low variability within the predicted neutral regions. Further, we validated sequencing-based CNV predictions by two independent experiment approaches including aCGH and qPCR. We performed 11 pairwise aCGH experiments using RJF as the reference and all others as the test samples. Considering that we estimated the CN of selected individuals with respect to reference genome which cannot be used for the aCGH reference sample, we calculated the predicted log_2_ CN ratios for the 11 test samples against RJF to make the CN values comparable with the aCGH results, which was designated as digital aCGH approach [12, 38]. We focused on the autosomal CNVs to avoid the impact of gender and unassigned linkage groups. For pairwise samples (each of the 11 test samples and RJF), there were two types of CNV events, i.e., overlapping and unique segments. For the overlapping CNV segments, we first split them into non-overlapping subsegments. And then we estimated the CN of each subsegment and unique segment longer than 1,000 bp for each of the two pairwise samples, and divided the copy number estimates of the test sample by that of RJF and calculated log_2_ CN ratios as digital aCGH values. Then we compared the digital values with aCGH probe log_2_ ratios which were defined as the average of all probes log_2_ ratio values in the corresponding segments. We performed a simple linear regression analysis to assess the correlation between the two values. The Pearson’s correlation coefficient (r) ranged from 0.435 in SK to 0.755 in DX (Figure 2 and Additional file 5: Figure S2), with an average of 0.647. BY (0.502), SK (0.435) and WR (0.491) showed lower correlation close to 0.500, and we found the mean of all probes log_2_ ratio values in the three aforementioned individuals were 1.05, 0.85 and 1.05 respectively, which were larger than the values of others that were close to zero.

In addition, we randomly chose 15 predicted CNVRs representing different types and frequencies for qPCR assays, and tested all 12 samples for each CNVR. Two distinct pairs of primers were designed for each predicted CNVR (Additional file 6: Table S4). The proportion of confirmed positive samples (positive predictive value) varied from 50 to 100%, with an average of 91.71%. However, some negative samples were also confirmed to contain CNVs, and the false negative rate varied from 0 to 60%, with an average of 22.43%. We illustrated the qPCR results for three confirmed CNVRs of different types (gain, loss and both) (Additional file 7: Figure S3).

### Copy number polymorphic genes

**Figure 3:**
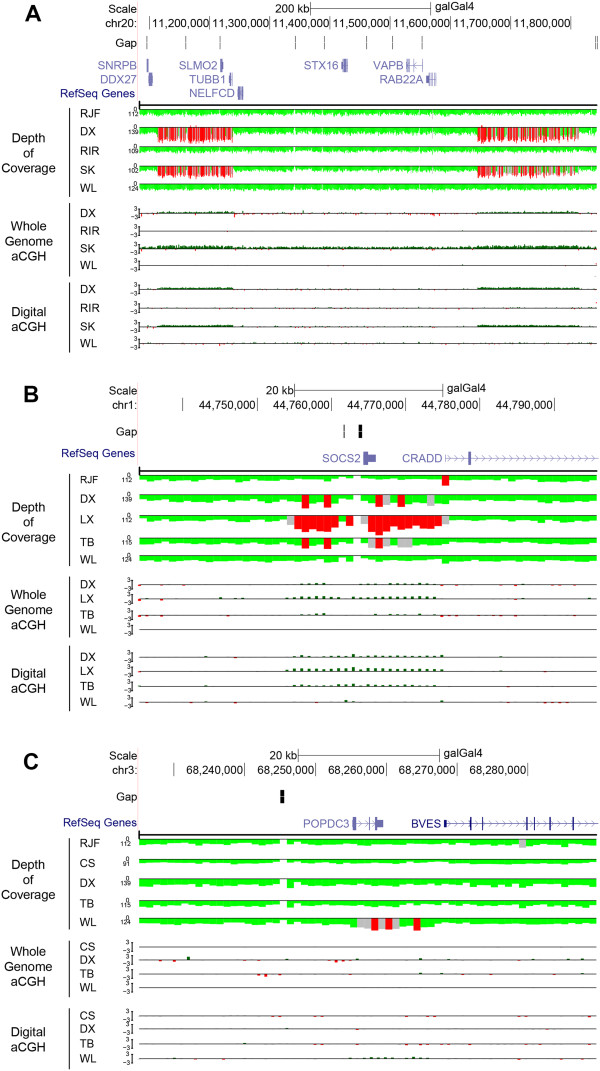
**Visual examination by read depth, whole-genome aCGH and digital aCGH around three loci for five representative chicken genomes.** The uppermost gene image is generated with the UCSC Genome Browser ( http://genome.ucsc.edu/) using the galGal4 assembly. The track below the gene region is depth of coverage for all five individual genomes. Red indicates regions of excess read depth (> mean +3 × STDEV), whereas gray indicates intermediate read depth (mean +2 × STDEV < × < mean +3 × STDEV), and green indicates normal read depth (mean ± 2 × STDEV). All read depth values based on 1 kb non-overlapping windows are corrected by GC content. Whole-genome aCGH and digital aCGH values are depicted as the red-green histograms and correspond to a gain colored in green (>0.5), a loss colored in red (<-0.5) and normal status colored in gray (-0.5 < × <0.5). **(A)** Two previously reported CNVs (chr20: 11,111,401-11,238,900 and chr20: 11,651,801-11,822,900) associated with dermal hyperpigmentation. The DX and SK genomes show two additional copies of the two regions compared with RJF, and are also validated by whole-genome aCGH. **(B)** A higher copy number increase for the *SOCS2* locus (chr1: 44,764,280-44,765,955) is predicted in LX than in other individuals. **(C)** The *POPDC3* gene (chr3: 68,255,196-68,259,535) is predicted to be duplicated status only in WL.

We obtained 6,086 non-redundant RefSeq gene transcripts retrieved from the UCSC Genome Browser and estimated the copy number values of all genes in different individuals by CNVnator. A total of 2,216 (36.4%) genes overlapped with 2,214 (25.0%) predicted CNVRs. Among them, 537 genes were found to be completely covered by CNVRs. The overlapping genes were found not to be highly duplicated sequences, and the maximum copy number estimates was only 12.0. We examined the 25 most variable genes according to the STDEV of their copy number estimates in different individuals (Additional file 8: Table S5), and found that these genes were mainly involved in immune response and keratin formation. It should be noted that the keratin gene families were detected to have large CN values and variance. Two significant CNVRs associated with dermal hyperpigmentation were located on chr20 at positions 11,217,001 to 11,272,200 (CNVR7962) and 11,651,801 to 11,822,900 (CNVR7968), respectively, which had already been described in detail in a previous study [30], and the distance between the two loci was 379.6 kb. *SLMO2* and *TUBB1* were completely covered by the first region which was predicted to be about twice as many copies in DX and SK as in other individuals (Figure 3A and Additional file 9: Figure S4a). The functional gene *EDN3* (endothelin 3) is not archived because the predicted gene is not available for UCSC RefSeq database. We found that only BY had this CNVR while SK and DX as two typical breeds with dermal hyperpigmentation did not. So we further checked the raw results before removing CNVs overlapping with gaps. Two nearly identical CNVs comprising two gaps (>100 bp) were found, one at positions 11,111,501 to 11,238,600 in DX and the other at positions 11,111,401 to 11,238,900 in SK, which were also confirmed by our whole genome aCGH experiments (Figure 3A and Additional file 9: Figure S4a). The distance between the raw CNVR and the second region (CNVR7968) was 412.9 kb, which perfectly supported the reported results [30]. Conversely, the first CNVR in BY (11,217,001 to 11,272,200) showing normal skin color does not contain the *EDN3* gene (11,148,025 to 11,160,484), which also provides evidence that the *EDN3* with copy number polymorphism is the causal mutation resulting in dermal hyperpigmentation. Another previously identified CNVR involving the *PRLR* (prolactin receptor) gene on chrZ [31] was also detected in our study, and the copy number estimates of *PRLR* in WC and WL were twice as many as in other individuals. The sex-linked *K* allele containing two copies of *PRLR* in females is associated with the late feathering and used widely for sexing hatchlings. Our sequencing-based and qPCR results showed that WC and WL should exhibit the late feathering phenotype, which were supported by the actual phenotype record.

**Figure 4:**
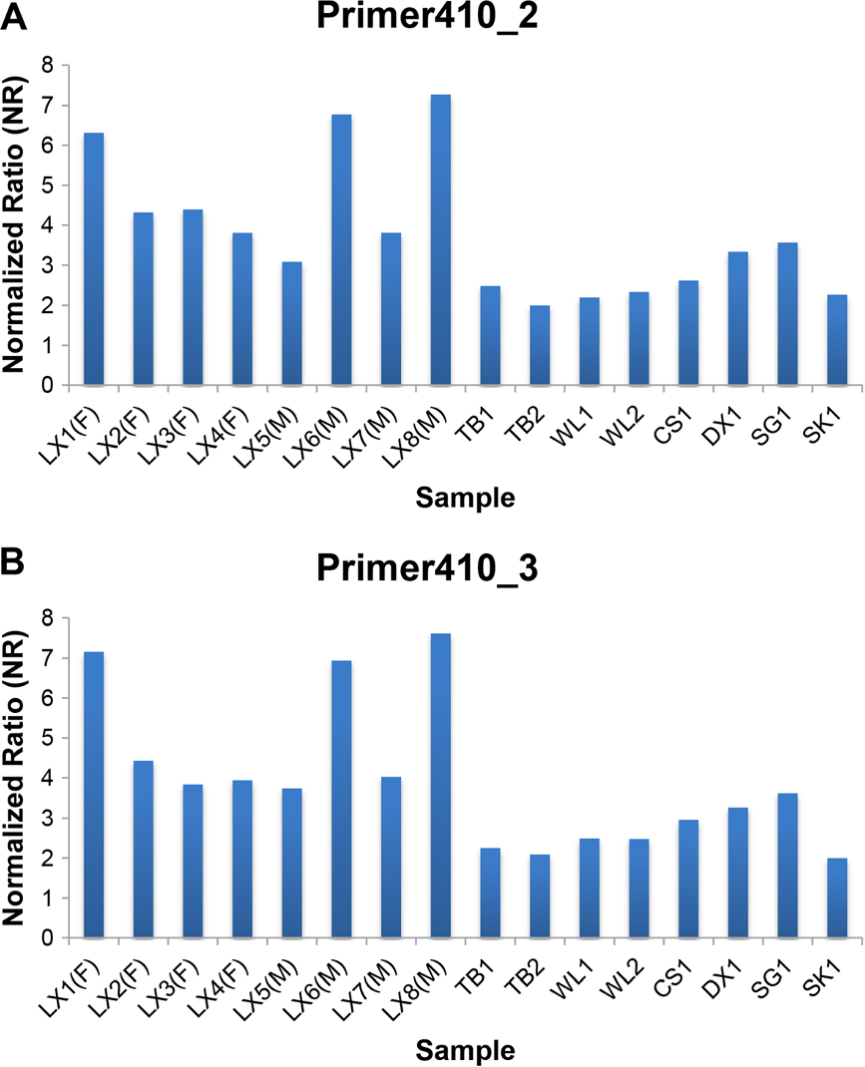
**Validation of CNVR410 by qPCR in another 16 chickens.** X-axis represents all 16 samples and Y-axis represents normalized ratios (NR) estimated by qPCR. NR around 2 indicates normal status (2 copies), NR around 0 or 1 indicates loss status (0 copies or 1 copy), and NR around 3 or more indicates gain status (3 or more copies). **(A)** qPCR results confirmed by primer410_2. **(B)** qPCR results confirmed by primer410_3.

In addition, we found that some genes related to the host immune and inflammatory response had CNVR overlaps, like *CD8A*, *FZD6*, *LIMS1*, *TNFSF13B* and some MHC-related genes associated with Marek’s disease (MD). The *SOCS2* involving in the regulation of bone growth and density was predicted to have the largest CN value in LX (n =6.4), while DX (n =3.0) and TB (n =3.6) also possessed the duplicated sequences in this locus compared with the neutral regions in other individuals (Figure 3B and Additional file 9: Figure S4b). LX represents a characteristic breed for cockfighting in which bone strength is an essential feature for selection. To validate the highly duplicated sequence (CNVR410) found only in LX, we selected another 16 individuals, i.e., eight LX (four males and four females) and other eight females consisting of one CS, one DX, one SG, one SK, two TB and two WL, to perform qPCR experiments using the same two pairs of primers listed in Additional file 6: Table S4. Two qPCR results demonstrated the copy number estimate of almost each LX was larger than the others (Figure 4), and the average copy number estimates (5.0 and 5.2 for two pairs of primers, respectively) of all LX were significant larger than those (2.6 and 2.6) in other individuals using the two-sample *t*-test (*P*-value =0.003 and 0.001). Additionally, other identified CNV-gene overlaps could be potentially responsible for certain economic traits, as these genes were involved in lipid metabolism, muscle development and protein secretion process. For example, our results suggested higher copy number for the *POPDC3* gene in WL (n =4.2) than in the other 11 genomes (n =2.3) (Figure 3C and Additional file 9: Figure S4c). Similarly, the WL genome showed the greatest number of *AVR2* copies (n =2.0) on chrZ compared with the others (n =1.1). Two promising genes involving in lipid metabolism, *AP2M1* and *LBFABP*, were identified as the largest copy number (n =3.0 and 3.2) in meat-type chicken (CS) compared with those in the others.

### Heatmap visualization

**Figure 5:**
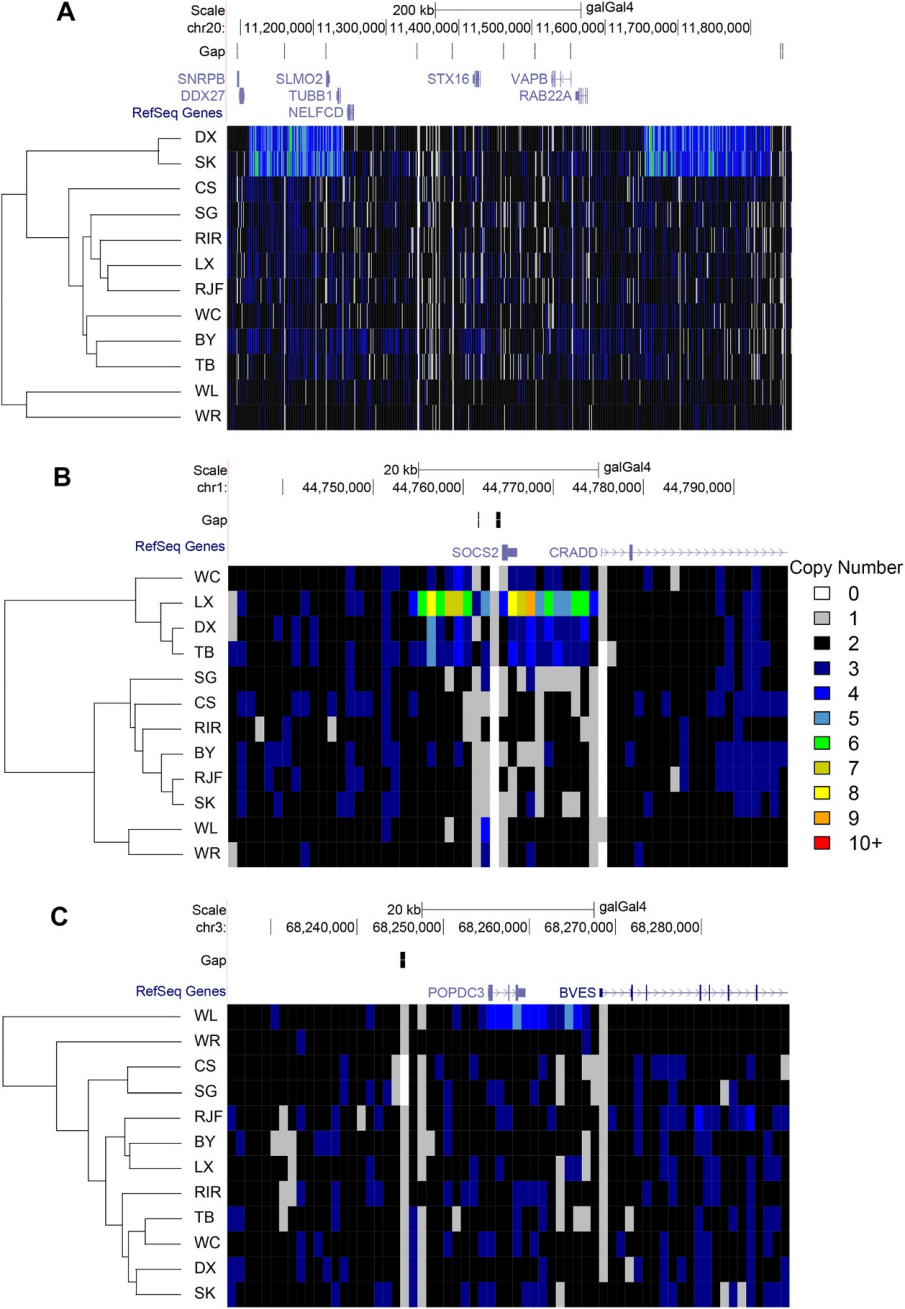
**Hierarchical clustered heatmaps of preselected genetic loci for 12 chicken genomes.** Every block in the heatmap indicates estimated CN values of 1 kb non-overlapping windows in the preselected region. These heatmaps are generated from hierarchical cluster analysis using Pearson’s correlation coefficient of the CN values. The colors for each bar denote different copy number (CN). **(A)** DX and SK which are predicted to be doubled within dermal hyperpigmentation loci are clustered together. **(B)** Upstream and downstream of the *SOCS2* locus reveal higher CN values in DX, TB and WC especially LX. **(C)** WL shows specific expansion in the *POPDC3* locus and is split into a separate clade.

We performed a hierarchical clustering analysis and generated heatmaps based on Pearson’s correlation coefficient using the CN values of selected gene loci, in order to infer the potential relationship of selected genes among 12 individuals. The loci encompassing *SLMO2* and *TUBB1* in DX and SK were found to be highly duplicated regions and the two individuals were clustered into one group (Figure 5A). Another promising gene, *SOCS2*, was also confirmed for the difference in copy number between LX and the others (Figure 5B). Meanwhile, WL showed specific expansion in the *POPDC3* locus and was split into a separate clade (Figure 5C).

### Gene content and QTL analysis of CNVRs

A total of 2,216 RefSeq genes overlapped with putative CNVRs. Then, we performed gene ontology (GO) and Kyoto Encyclopedia of Genes and Genomes (KEGG) pathway analysis for these genes. The GO analysis revealed 646 GO terms, of which 175 were statistically significant after Benjamini correction (Additional file 10: Table S6). The significant GO terms were mainly involved in positive regulation of macromolecule metabolic process and gene expression, plasma membrane, protein localization, enzyme binding, response to oxidative stress and immune system development. The KEGG pathway analysis indicated that these genes were overrepresented in nine pathways, but none of which was significant after Benjamini correction. According to our artificial QTL filtering criteria, we identified 595 high-confidence QTLs in total, of which 300 (50.4%) were found to overlap with 560 (6.3%) CNVRs (Additional file 11: Table S7). These QTLs were mainly involved in production and health traits, such as growth, body weight, abdominal fat weight, egg number and Marek’s disease-related traits.

## Discussion

This study performed genome-wide CNV detection, determined absolute copy number and constructed the first individualized chicken CNV map. The NGS technology and RD method employed in our work has advantages in both technology platform and genetic diversity compared with the eight previous reports [9, 32–34, 36, 39–41]. Because a significant fraction of CNVs falls into genomic regions not well-covered by microarrays, especially for SD regions lacking sufficient probes [16, 23], CNV as a major source of genetic variation is complementary to SNP and could account for a substantial part of missing heritability [14]. Most CNV studies to date have been discovery studies rather than association studies, mainly due to the limitations of CNV resolution and genotyping in each individual [3]. The high-resolution individualized chicken CNV map based on extensive genetic diversity not only enriches the current genetic variation database but also encourages the future development of assays for accurately genotyping CNVs, enabling systematic exploration about CNV association studies similar to SNPs. In future, integration of CNVs with SNPs may be an effective and promising way to elucidate the causes of complex diseases and traits [16, 17].

### Genome-wide CNV landscape in the chicken genome

The number of CNVs and CNVRs in each individual varied greatly, and all individuals shared a small number of them, suggesting that a considerable proportion of CNVs likely segregated among 12 distant breeds [2, 34], therefore a larger population and multiple breeds are crucial to construct a more complete chicken CNV map. The high percentage of unique CNVRs could also be partly explained by the high recombination rate in the chicken genome (2.5-21 cM/Mb), because recombination-based mechanisms such as non-allelic homologous recombination (NAHR) are the major causes leading to CNVs [42]. Similarly, the high recombination rate may induce more denser CNVRs in microchromosomes [43]. These unique CNVRs may be recent events in evolution and contribute to breed-specific phenotype and performance [44]. Compared with the eight previous chicken CNV studies [9, 32–34, 36, 39–41], far more CNVRs both on average and in total were found. A total of 6,478 (86.0%) autosomal CNVRs with slightly smaller average size (10.6 kb) were novel, likely due to the higher resolution and sensitivity of NGS method than aCGH and SNP array. These novel CNVRs enrich significantly the published chicken CNV database. The low concordance between different studies results from the differences in technical issues, CNV calling algorithms as well as study populations [45], and can also indicate that numerous CNVs in the chicken genome are still expected to be discovered.

We found both maximum and mean copy number estimates of autosomal duplicated sequences in chickens were less than those in mammalians [2, 12], which might be related to the relatively smaller genome size (only one third of a typical mammalian genome) and the lower repetitive DNA content in the chicken genome [46]. In addition, both the counts and sizes of gain events were larger than losses because chromosomal deletion can lead to a variety of serious malformations and disorders and is subjected to purifying selection [13, 47]. In general, the length of chromosome is positively correlated with the number of CNVRs. The chr16 (a microchromosome) was found to have the second densest CNVRs, possibly owing to the highly variable major histocompatibility complex (MHC) regions and higher recombination rate [48], which also results in repeatedly finding the same CNVRs on chr16 among different studies.

### Quality assessment and experimental validation

It is generally believed that the CN of neutral regions is between 1.5 and 2.5 [25] and the mean ± 2 × STDEV in our results corresponded closely to the hypothesis, which demonstrates that CNVnator has efficient performance on CNV detection and CN estimation and can generate most reliable results. For CNV quality assessment by aCGH, the positive correlation values between computational and experimental log_2_ CN ratios in our study were higher than the previous results [2], mainly owing to the aCGH platform with higher resolution in our analysis. The slightly low correlation coefficients in BY, SK and WR might disclose certain experimental noises and biases resulting in misgenotyping in corresponding aCGH experiments [16], and particularly highly duplicated regions and rare deletions [15, 25]. In addition, the average positive predicted value of the 15 chosen CNVRs was 91.71%, similar to some previous results in animals [7, 33, 45], suggesting that most of the positive samples detected by sequencing-based method are highly consistent with the qPCR experiments. We also estimated the false negative error rates as it is a common problem in CNV detection [7, 49], and the average percentage of false negative results was 22.43%. The discrepancies between NGS results and qPCR validation may be due to the negative impact of potential SNPs and small INDELs, which result in the reduced primer efficiency.

### Promising candidate genes covered by CNVRs

CNV is a significant source of genetic variation accounting for disease and phenotypic diversity, owing to the duplication or deletion of covered genes or regulation elements [4]. Our results showed that 36.4% RefSeq genes intersected with 25.0% predicted CNVRs. It is probable that CNVs, especially deletions, are located preferably in gene-poor regions [13, 47], because gene-rich CNVRs are more likely to be pathogenic than gene-poor CNVRs and these deleterious CNVRs would be removed by purifying selection [47, 50]. Meanwhile, the maximum CN of all genes covered by CNVRs was 12.0, suggesting again that the chicken genome has lower repetitive DNA content [46]. It is noted that nine out of the 25 most variable genes belong to four keratin subfamilies (claw, feather, feather-like and scale). In birds, skin appendages such as claws, scales, beaks and feathers are composed of beta (β) keratins and can prevent water loss and provide a barrier between the organism and external environment [51]. The avian keratin genes are significantly over-represented with respect to mammals [34, 48]. These highly variable keratin genes suggest the scenario for the evolution of the β-keratin gene family through gene duplication and divergence for their adaptive benefits [4, 51]. Additionally, the four subfamilies of β-keratin genes form a cluster on chr25, one of the more GC-rich chromosomes and containing a relatively larger number of minisatellites [51], which also result in high copy number of genes.

We validated two well-known causative genes with copy number polymorphism, *EDN3* [30] and *PRLR* [31], involved in dermal hyperpigmentation and late feathering, respectively. In our study, we used hierarchical clustering analysis based on CN content to visualize the potential relationship among 12 breeds. For example, the heatmap for dermal hyperpigmentation grouped DX and SK together, and both of which are distributed in the Jiangxi province of China, suggesting that DX and SK may have a close evolutionary relationship or purposely bred dermal hyperpigmentation into different strains. In addition, two reported copy number variable genes associated with Marek’s disease, namely *FZD6* (frizzled family receptor 6) and *LIMS1* (LIM and senescent cell antigen-like domains 1) [39, 52, 53] were also found in our results.

Furthermore, we also found some novel CNV-gene overlaps as potential candidates linked to some important traits. For example, the *SOCS2* (suppressor of cytokine signaling 2) is a member of the suppressor of cytokine signaling family, and the related proteins are implicated in the negative regulation of cytokine action through inhibition of the JAK/STAT pathway (Janus kinase/signal transducers and activators of transcription) [54]. Dual x-ray absorptiometry (DXA) analysis demonstrated that *SOCS2* inactivation resulted in reduced trabecular and cortical volumetric bone mineral density (BMD) in *SOCS2*-deficient mice [55]. We found that the *SOCS2* had higher CN (n =6.4) in LX than in other individuals, which is particularly interesting as the LX is known for cockfighting in which the chickens with higher BMD have advantage over others. The gene expansions were also supported by the heatmap. Additional qPCR experiments in 16 other individuals revealed that the increased copy number of *SOCS2* in LX was larger than others. We suspect that the copy number polymorphic locus is ubiquitous in the chicken genome, but the particularly high gene duplication in LX may be the result of the genetic effect of long-term artificial selection such as crossing between the individuals with stronger bone.

Additionally, the copy number estimates of *POPDC3* (popeye domain containing 3) in WL were found to be about twice as many as other individuals. The *POPDC3* gene belongs to the Popeye family encoding proteins with three potential transmembrane domains with a high degree of sequence conservation, and is preferentially expressed in heart and skeletal muscle cells as well as smooth muscle cells [56]. It has been reported that the expression of two Popeye family members was upregulated in the uterus of pregnant mice [57]. The uterus has been thought to be an important organ composed of smooth muscle and containing the shell gland in favor of depositing eggshell [58]. Considering that WL is the most prolific egg laying chicken due to the fact that it has been extensively bred for egg production, the duplication of the *POPDC3* gene may reveal the important differences in abilities like myometrium maturation and labor, protein secretion and eggshell formation between WL and other breeds.

Moreover, these enriched GO terms were mainly involved in cellular regulation and structure, various binding functions as well as stress and immune responses, which are consistent with several previous studies [9, 32–34], suggesting that the copy number variable genes may influence the responses to external stimuli and provide the mutational flexibility to adapt rapidly to changing selective pressures due to evolutionary adaption [59]. Most CNVRs also spanned some QTL regions, which indicated that these CNVRs may exert significant effects on traits of economic interest in chickens.

## Conclusions

In this study, we performed genome-wide CNV detection and estimated the absolute copy number of the corresponding genetic locus based on whole genome sequencing data of 12 chickens abundant in genetic diversity, and constructed the highest-resolution individualized chicken CNV map so far. A total of 8,840 CNVRs were identified, and most of them were novel variants which could enrich the current CNV database. The high CNVR confirmation rates by aCGH and qPCR suggested that sequencing-based method was more sensitive and efficient for CNV discovery. We detected 2,216 RefSeq genes overlapping with CNVRs, including genes involved in well-known phenotypes such as dermal hyperpigmentation and late feathering. In addition, some novel genes like *POPDC3* and *LBFABP* covered by CNVRs may play an important role in production traits, and the highly duplicated *SOCS2* may serve as an excellent candidate for bone mineral density. Our study lays the foundation for comprehensive understanding of copy number variation in the chicken genome and is beneficial to future association studies between CNV and important traits of chickens.

## Methods

### Sample collection and sequencing

We selected a total of 12 female chickens from different types and genetic sources representing modern chicken populations, i.e., a Red Jungle Fowl (RJF, the ancestor of domestic chickens), seven Chinese indigenous chickens including Beijing You (BY), Dongxiang (DX), Luxi Game (LX), Shouguang (SG), Silkie (SK), Tibetan (TB) and Wenchang (WC), and four commercial breeds including Cornish (CS), Rhode Island Red (RIR), White Leghorn (WL) and White Plymouth Rock (WR). The whole blood samples were collected from brachial veins by standard venepuncture along with regular quarantine inspection of the experimental station of China Agricultural University, and genomic DNA was isolated using the standard phenol/chloroform extraction method. Whole genome sequencing for all 12 individuals was performed on the HiSeq 2000 system (Illumina Inc., San Diego, CA, USA). Two genomic DNA libraries of 500 bp insert size per individual were constructed and sequenced with 100 bp paired-end reads, and each library dataset was generated with five-fold coverage depth. Library preparation and all Illumina runs were performed as the standard manufacturer’s protocols.

### Quality control and sequence alignment

For ensuring high-quality data, we used NGS QC Toolkit with default parameters to perform quality control of raw sequencing data, mainly by removing low-quality reads and reads containing primer/adaptor contamination [60]. All high-quality Illumina sequence reads were aligned against the galGal4 assembly by using the Burrows-Wheeler Aligner (BWA) program [61] with default parameters. The draft genome sequence was retrieved from the UCSC website ( http://hgdownload.soe.ucsc.edu/goldenPath/galGal4/bigZips/). During the construction of a genomic library, Illumina platform was likely to generate some duplicate reads named ‘PCR and optical duplicates’ which imposed negative impact on the downstream analysis. So we first used SAMtools [62] to convert the .sam files of different libraries belonging to the same individual to .bam files and sort and merge them, followed by removal of potential PCR duplicates using Picard ( http://broadinstitute.github.io/picard/).

### CNV detection

Following the above filtering steps, the resulting .bam files were utilized for CNV calling and genotyping, post-processing was performed using CNVnator software based on RD method as previously described [25]. CNVnator firstly calculated the counts of mapped reads within user specified non-overlapping bins of equal size as the RD signal, and then adjusted the signal in consideration of the potential correlation between RD signal and GC content of the underlying genomic sequence. The mean-shift algorithm was employed to segment the signal with presumably different underlying CN. Then CNVs were predicted by applying statistical significance tests to the segments. A more detailed description about this method could be found in the CNVnator paper [25]. We ran CNVnator with a bin size of 100 bp for our data. CNV calls were filtered using stringent criteria including *P*-value <0.01 and size >1 kb, and calls with >50% of q0 (zero mapping quality) reads within the CNV regions were removed (q0 filter). All CNV calls overlapping with gaps in the reference genome were excluded from consideration. For unlocalized and unplaced chromosomes (chrN_random and chrun_random in UCSC, chrUn), we removed them for further analysis due to the shorter length of the chrUn contigs and mapping ambiguity of chrUn sequence reads. Meanwhile, we performed genotyping of all 5 kb non-overlapping windows which did not overlap with putative CNVs and gaps on autosomes. In order to compare our results with previous studies, we converted all autosomal CNVRs from galGal4 to galGal3 using the UCSC liftOver tool [63].

### Array CGH for assessing genome-wide CNVs

We conducted CNV consistency evaluation using two similar whole genome tiling arrays based on galGal4 2011 build. One of them is the NimbleGen aCGH (Madison, WI, USA), a custom-designed 3*1.4 M array containing a total of 1,425,178 50-75mer probes with the mean and median interval of 734 bp and 700 bp. The other is the Agilent custom-designed 1*1.0 M array (Agilent Technology Inc., CA, USA), with the mean and median probe spacing of 1,056 bp and 1,050 bp. It should be noted that the average physical distance of the closest SNP probes between two arrays was 262.6 bp and 95.2% distance intervals were shorter than 500 bp. Meanwhile we only analyzed raw aCGH log_2_ ratio values instead of processed/normalized data. These cases could ensure reasonable explanation for our results although using different arrays. All processing steps like DNA labeling (Cy3 for samples and Cy5 for references), array hybridization, data normalization and scanning analysis were performed following standard procedure. In each aCGH experiment, we chose the RJF as the same reference sample.

### Quantitative PCR confirmation

We also performed qPCR confirmation of 15 CNVRs chosen from the CNVRs detected by CNVnator. Most chosen CNVRs have not been reported in the previous studies and are also adjacent to annotated genes. Two distinct pairs of PCR primers were designed to target each CNVR using Primer5.0 software for the uncertainty of the CNVR boundaries. Furthermore, the UCSC In-Silico PCR tool was used for in silico analysis of primers specificity and sensitivity [63]. The *PCCA* gene which was previously identified as a non-CNV locus was chosen as the control region [40]. Quality control of all primer sets was evaluated using an 8-point standard curve in duplicate to ensure the similar amplification efficiencies between target and control primers. All qPCR experiments were conducted on the ABI Prism 7500 sequence detection system (Applied Biosystems group) using SYBR green chemistry in triplicate reactions, each with a reaction volume of 15 μl in a 96-well plate. The condition for thermal cycle was as follows: 1 cycle of pre-incubation at 50°C for 2 min and 95°C for 10 min, 40 cycles of amplification (95°C for 15 s and 60°C for 1 min). We used the formula 2^(1 - ΔΔCt)^ method to calculate the relative copy number for each test region. The cycle threshold (Ct) value of each test sample was first normalized against the control region, and then the ΔCt value was calculated between the test sample and a preselected reference sample predicted with normal copy number status by CNVnator. The golden standard of each diploid CNV was generally considered to have two copies for autosomes or one copy when the locus was on Z chromosome (chrZ) of a female in chickens.

### Gene contents and functional annotation

The RefSeq gene list was retrieved from the UCSC RefSeq database [63]. All miRNA genes were excluded because the nucleotide sequences were too short to estimate reliable copy number. We analyzed the proportion of the RefSeq genes overlapping with putative CNVRs and performed CN estimates for all 6,086 non-redundant RefSeq gene transcripts. In addition, to provide insight into the functional enrichment of the RefSeq genes covered by CNVRs, we performed Gene Ontology (GO) functional annotation and Kyoto Encyclopedia of Genes and Genomes (KEGG) pathway analysis employing the web-accessible program DAVID [64]. Statistical significance was accessed by using a modified Fisher’s exact test and Benjamini correction for multiple testing (*P*-value <0.05). We also compared the CNVRs identified in this study with the reported QTLs obtained from the chicken QTL database [65]. We focused on the QTLs with confidence interval less than 10 Mb and considered those QTLs with overlapped confidence intervals greater than 50% as the same QTL [45], because the QTL confidence intervals were too large to be used efficiently in the post-processing.

### Hierarchical cluster analysis

We used the heatmap.2() function of the gplots package ( http://cran.r-project.org/web/packages/gplots/index.html) to generate heatmap figures. We first specified the regions extending 30 kb on each side of interested genes and used the estimated CN values of 1 kb non-overlapping windows in each individual for post-analysis, mainly considering that the regulatory elements may be included in the upstream or downstream of a gene. No reordering of those windows representing corresponding chromosome locations in the heatmap was made for the sake of clarity. The Pearson’s correlation coefficient of the CN values was used as the distance measure of the agglomerative hierarchical clustering with average linkage, and to generate hierarchical cluster dendrograms.

### Availability of supporting data

All raw sequence data has been deposited in NCBI Sequence Read Achieve (SRA) under the Bioproject number PRJNA232548. The experiment numbers for the 12 chickens are SRX408161-SRX408172. All aCGH data have been submitted to the NCBI Gene Expression Omnibus (GEO) ( http://www.ncbi.nlm.nih.gov/geo/) under accession number GSE54119.

## Electronic supplementary material

Additional file 1: Table S1: Summary of identified CNVs and CNVRs in the 12 chicken genomes. (XLSX 1 MB)

Additional file 2: Figure S1: Individualized chicken CNV map in the chicken genome. The horizontal black lines represent the draft chicken genome (UCSC version galGal4). Tracks under the chromosomes indicate corresponding CNV status of all individuals kept in the alphabetical order from top to bottom, for BY, CS, DX, LX, RIR, RJF, SG, SK, TB, WC, WL and WR. Merged CNVRs from all individuals are depicted above chromosomes. The colors for each bar denote different copy number (CN) in CNV legend and different types of CNVRs. The downmost axis shows the chromosome, CNV and CNVR coordinates. Left-hand chromosomes are ordered from left to right, and the right-hands are just reversed. (PDF 180 KB)

Additional file 3: Table S2: General statistics of the CNVRs on each chromosome. (XLSX 13 KB)

Additional file 4: Table S3: Summary of novel or reported CNVRs on autosomes. (XLSX 325 KB)

Additional file 5: Figure S2: Correlation between digital aCGH and whole-genome aCGH among nine individuals compared with Red Jungle Fowl (RJF). RJF is selected as the reference sample in each aCGH experiment. Digital aCGH values are estimated using calculated log_2_ CN ratios in which CN are estimated for identified CNV segments of nine individuals and divided by the corresponding CN of RJF. Whole genome aCGH values are defined as the average of all probes log_2_ ratio values in the same segments as the digital aCGH. (PDF 7 MB)

Additional file 6: Table S4: Primers information and confirmation results of the 15 chosen CNVRs by qPCR analysis. (XLSX 20 KB)

Additional file 7: Figure S3: Illustrating of qPCR confirmation results for three selected CNVRs of different types. X-axis represents all 12 samples and Y-axis represents normalized ratios (NR) estimated by qPCR. NR around 2 indicates normal status (2 copies), NR around 0 or 1 indicates loss status (0 copies or 1 copy), and NR around 3 or more indicates gain status (3 or more copies). (A) Results for a gain status of CNVR3588. (B) Results for a loss status of CNVR6695. (C) Results for a both status of CNVR410. (PDF 63 KB)

Additional file 8: Table S5: The detailed features of RefSeq genes completely or partial overlapped with CNVRs. (XLSX 592 KB)

Additional file 9: Figure S4: Visual examination by read depth, whole-genome aCGH and digital aCGH around three loci for 12 chicken genomes. The uppermost gene image is generated with the UCSC Genome Browser ( http://genome.ucsc.edu/) using the galGal4 assembly. The track below the gene region is depth of coverage for all 12 individual genomes. Red indicates regions of excess read depth (> mean +3 × STDEV), whereas gray indicates intermediate read depth (mean +2 × STDEV < × < mean +3 × STDEV), and green indicates normal read depth (mean ± 2 × STDEV). All read depth values based on 1 kb non-overlapping windows are corrected by GC content. Whole-genome aCGH and digital aCGH values are depicted as the red-green histograms and correspond to a gain colored in green (>0.5), a loss colored in red (<-0.5) and normal status colored in gray (-0.5 < x <0.5). (A) Two previously reported CNVs (chr20: 11,111,401-11,238,900 and chr20: 11,651,801-11,822,900) associated with dermal hyperpigmentation. The DX and SK genomes show two additional copies of the two regions compared with RJF, and are also validated by whole-genome aCGH. (B) A higher copy number increase for the *SOCS2* locus (chr1: 44,764,280-44,765,955) is predicted in LX than in other individuals. (C) The *POPDC3* gene (chr3: 68,255,196-68,259,535) is predicted to be duplicated status only in WL. (PDF 2 MB)

Additional file 10: Table S6: Functional enrichment of GO and KEGG pathway analysis of RefSeq genes covered by CNVRs. (XLSX 54 KB)

Additional file 11: Table S7: The overlap information of QTLs and CNVRs across the chicken genome. (XLSX 70 KB)

## Abbreviations

CNV: Copy number variation
CNVR: Copy number variation region
aCGH: array comparative genomic hybridization
qPCR: quantitative polymerase chain reaction
NAHR: Non-allelic homologous recombination
NHEJ: Non-homologous end joining
FoSTes: Fork stalling and template switching
SD: Segmental duplication
GWAS: Genome-wide association studies
RD: Read depth
SNP: Single nucleotide polymorphism
NGS: Next-generation sequencing
GO: Gene ontology
KEGG: Kyoto encyclopedia of genes and genomes
QTL: Quantitative trait loci
MHC: Major histocompatibility complex
MD: Marek’s disease
BMD: Bone mineral density.
