# Supplementary figures and images for: Genome-wide patterns of copy number variation in the diversified chicken genomes using next-generation sequencing
Source: BMC Genomics. 2014 Nov 7;15(1):962. doi: 10.1186/1471-2164-15-962 (PMC4239369; doi:10.1186/1471-2164-15-962)

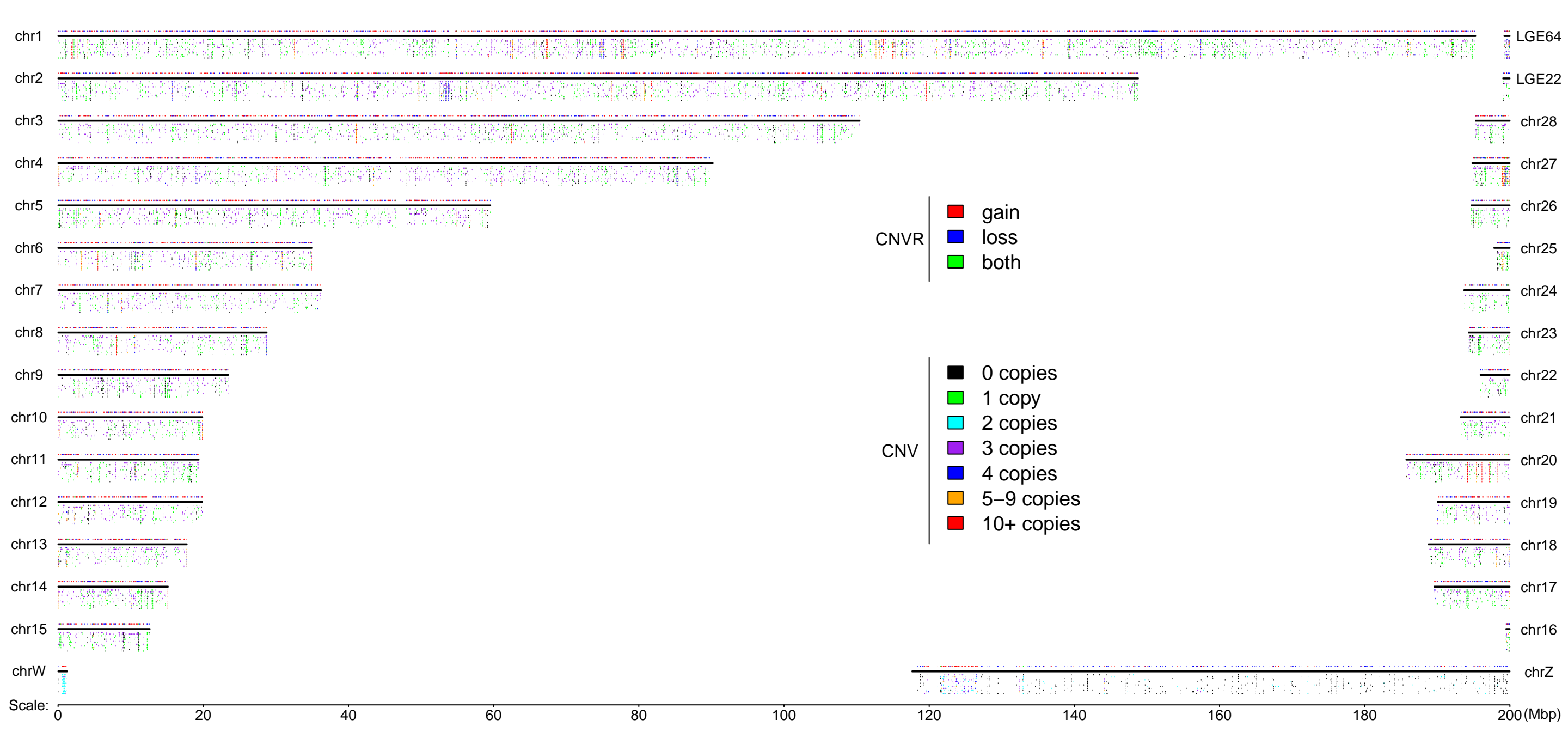

Supplement: Supplementary file 2 — Additional file 2: Figure S1: Individualized chicken CNV map in the chicken genome. The horizontal black lines represent the draft chicken genome (UCSC version galGal4). Tracks under the chromosomes indicate corresponding CNV status of all individuals kept in the alphabetical order from top to bottom, for BY, CS, DX, LX, RIR, RJF, SG, SK, TB, WC, WL and WR. Merged CNVRs from all individuals are depicted above chromosomes. The colors for each bar denote different copy number (CN) in CNV legend and different types of CNVRs. The downmost axis shows the chromosome, CNV and CNVR coordinates. Left-hand chromosomes are ordered from left to right, and the right-hands are just reversed. (PDF 180 KB) [file 12864_2014_6663_MOESM2_ESM.pdf]

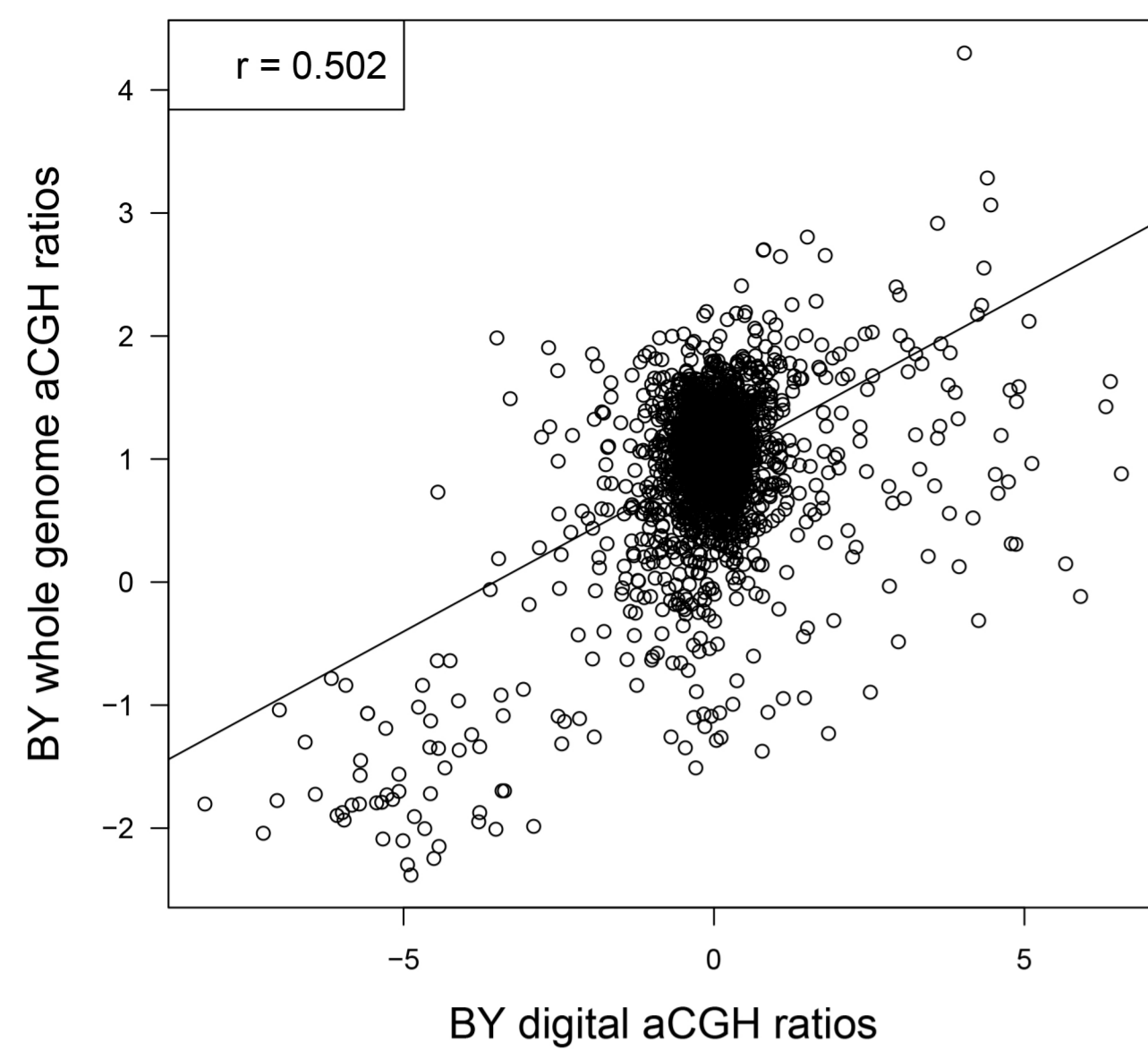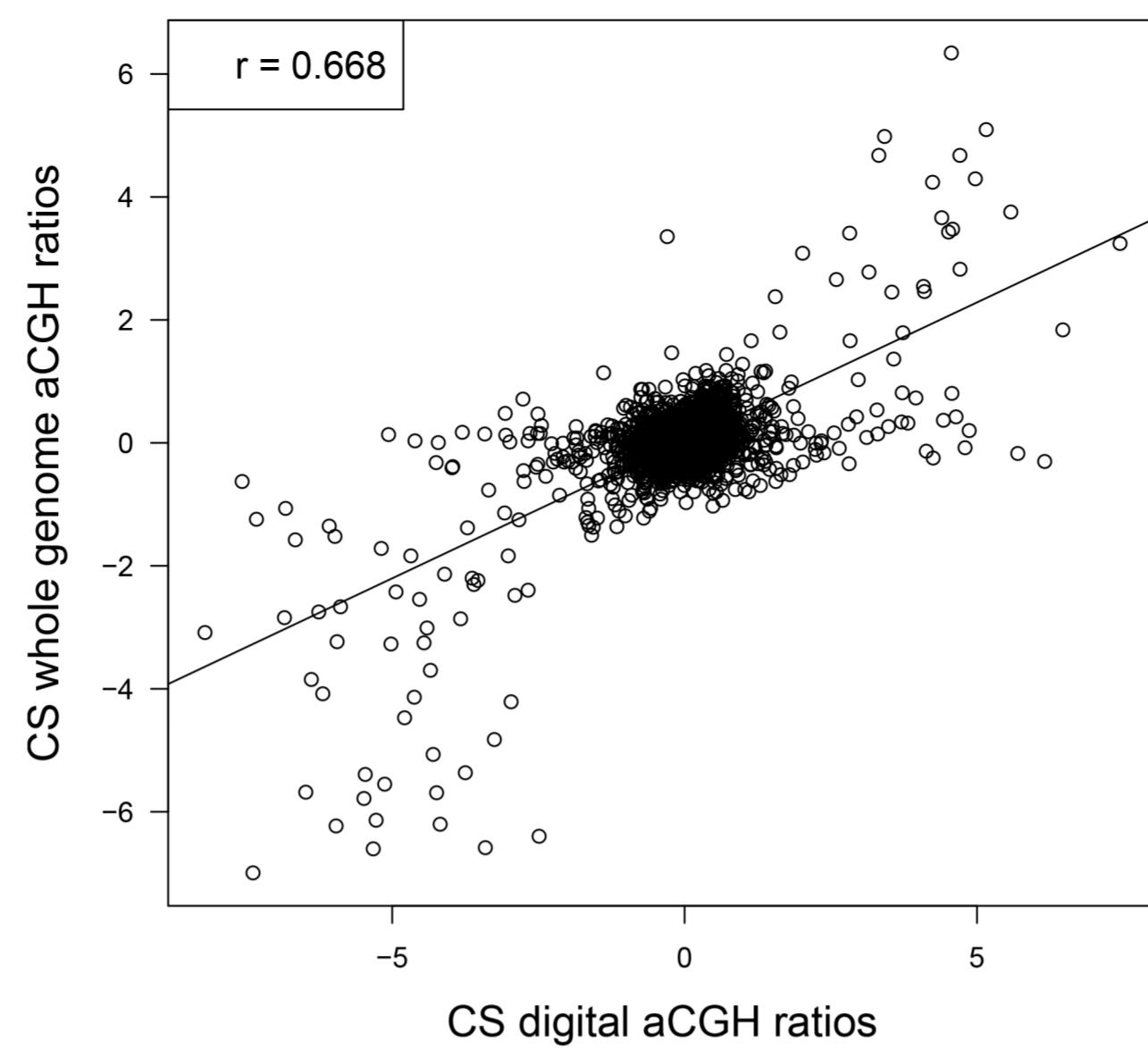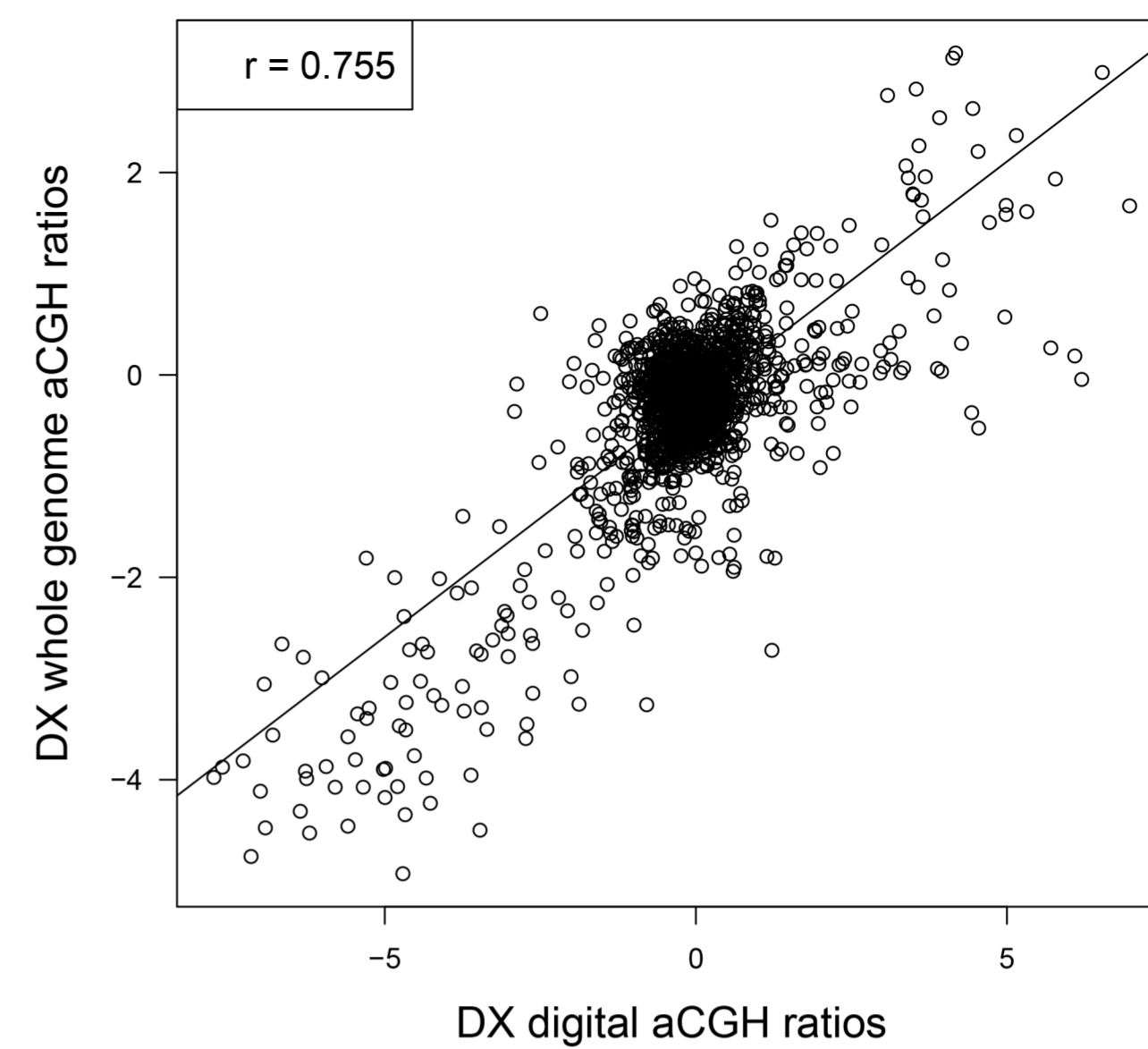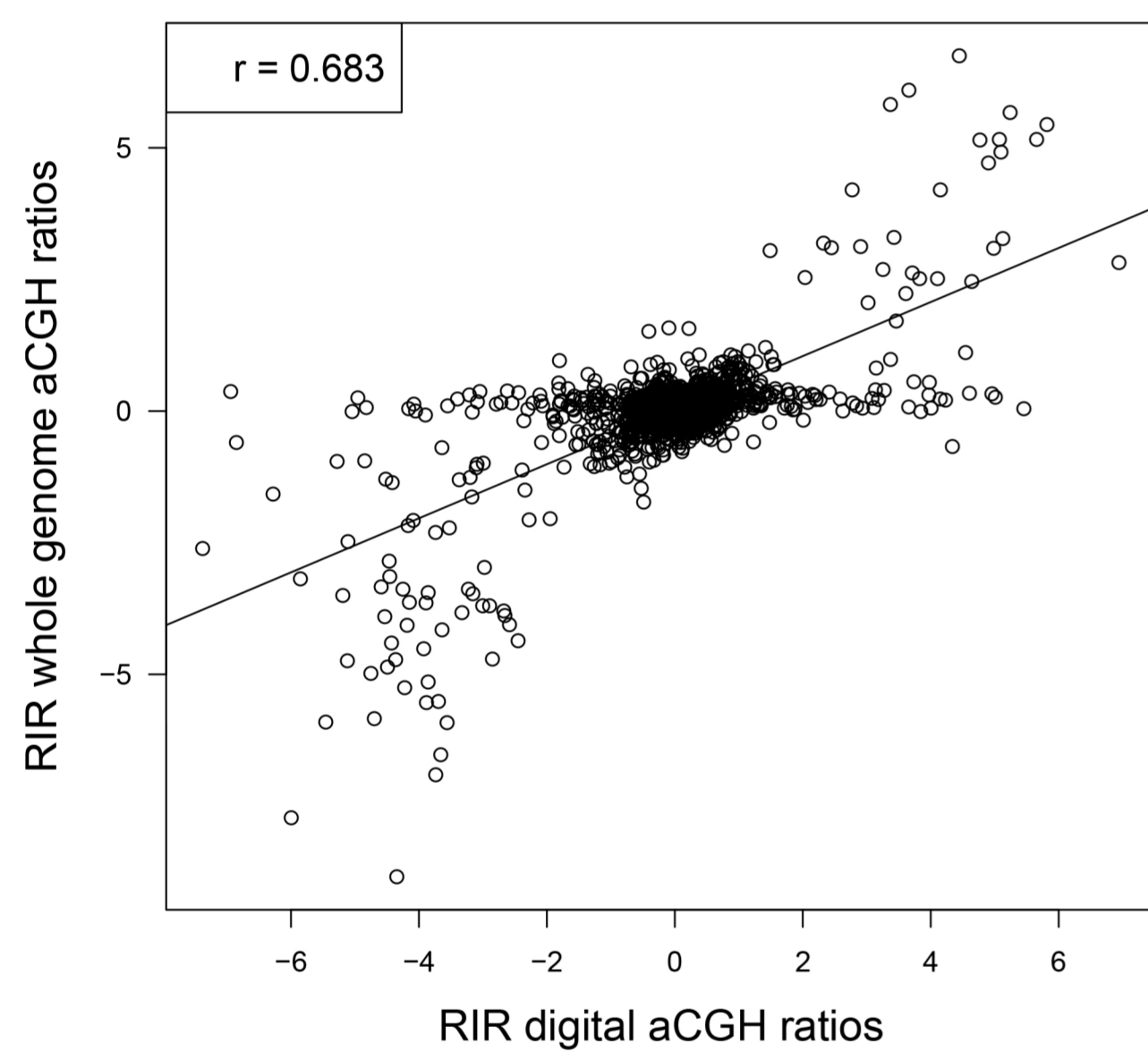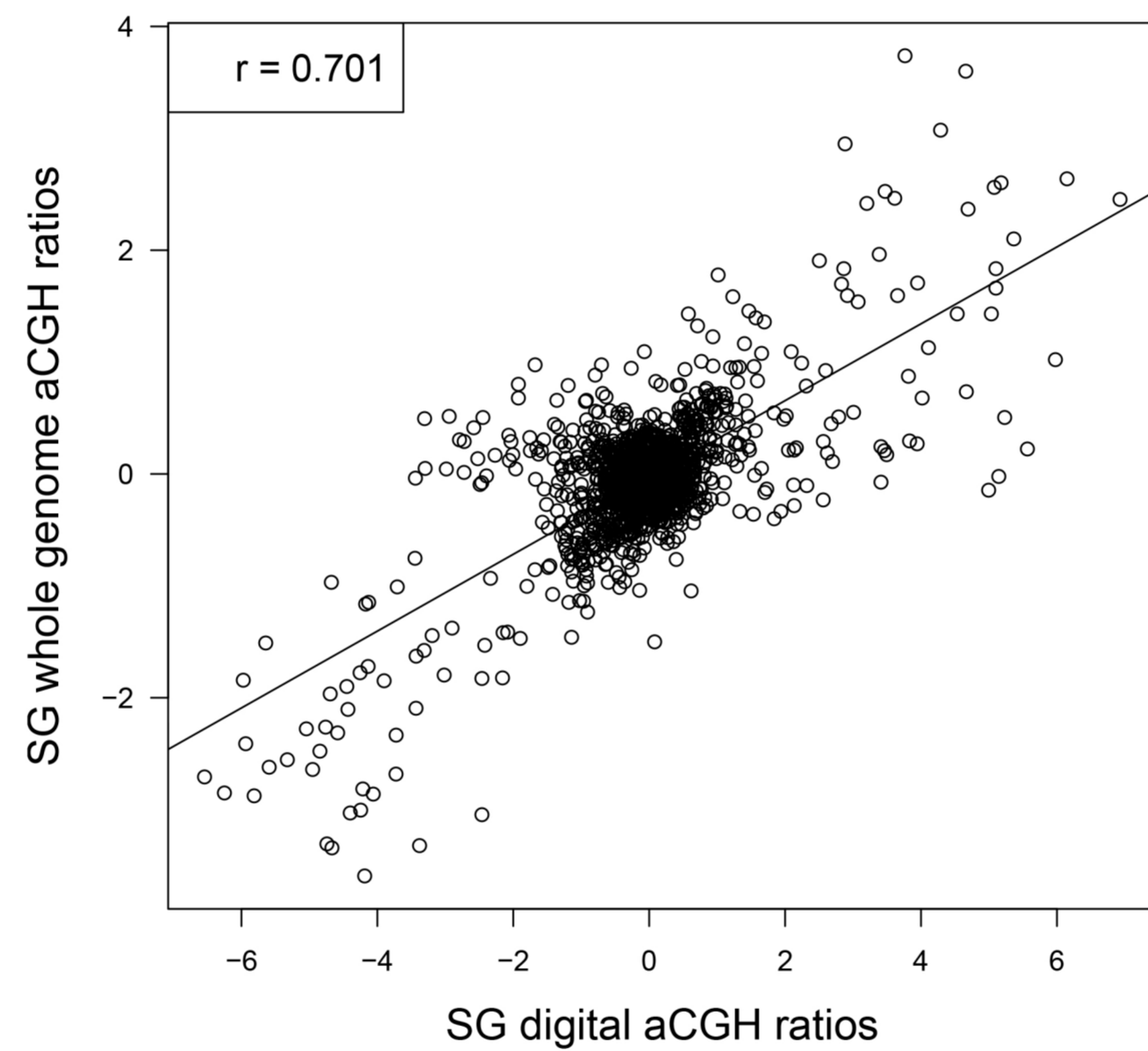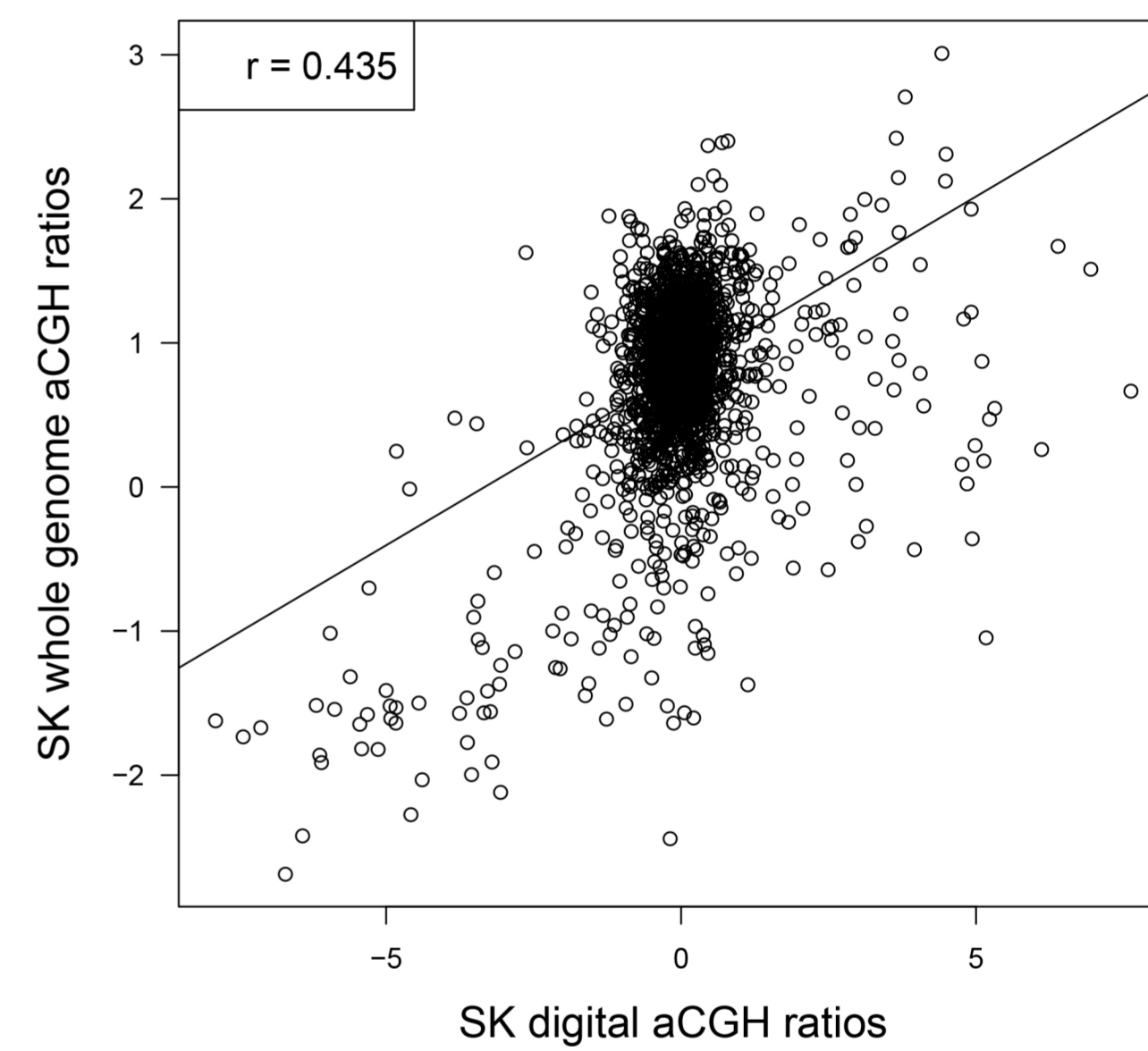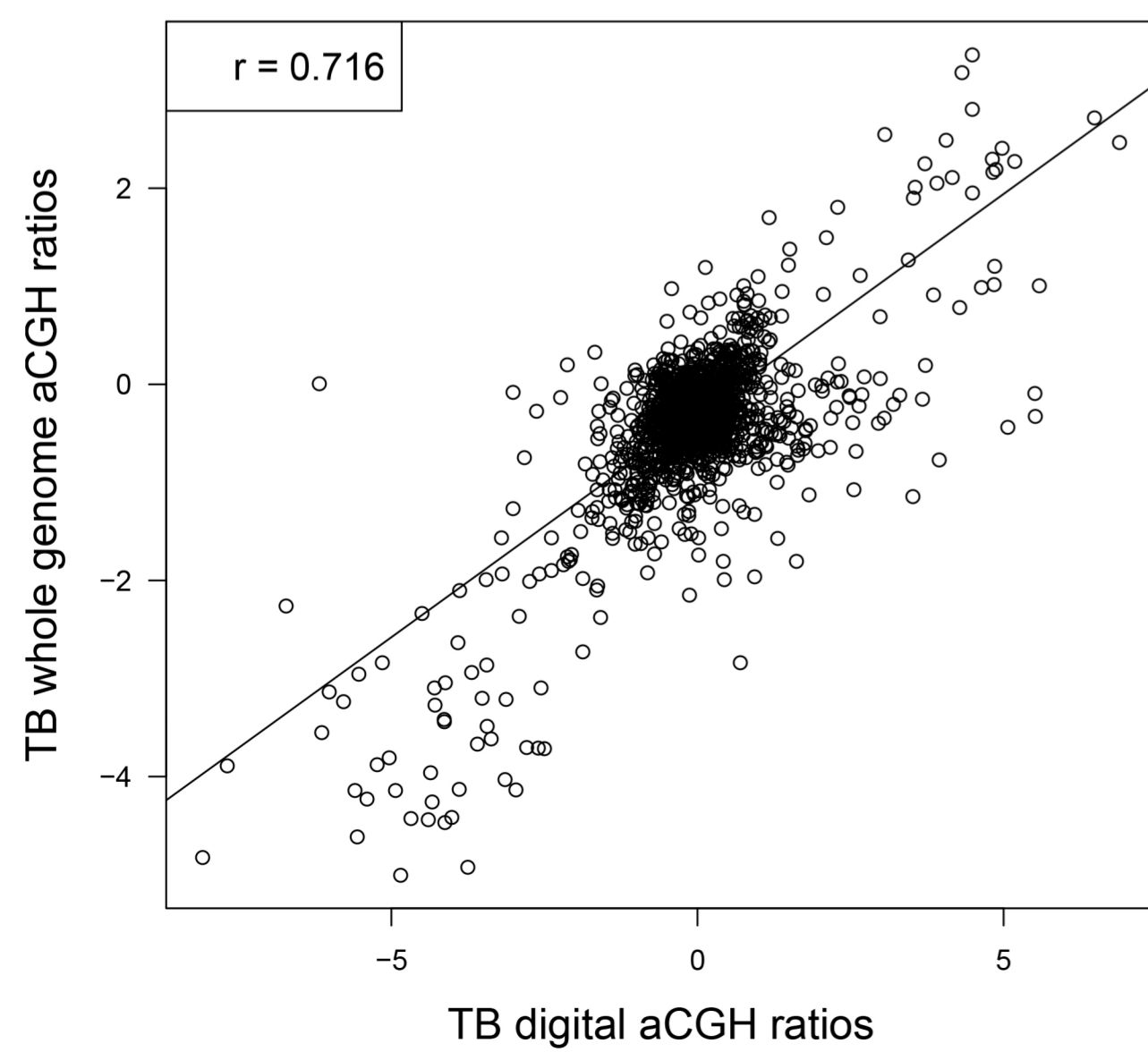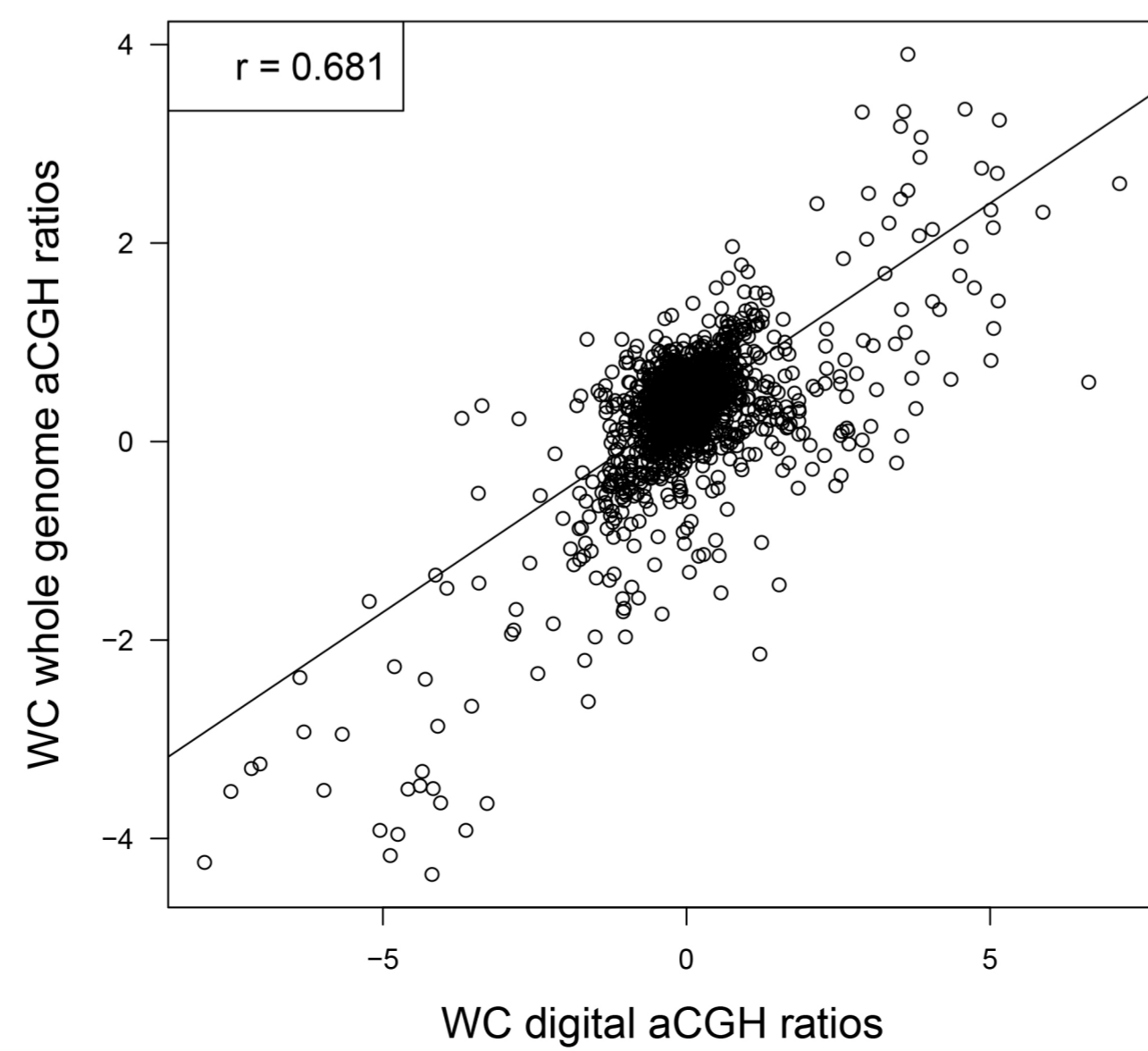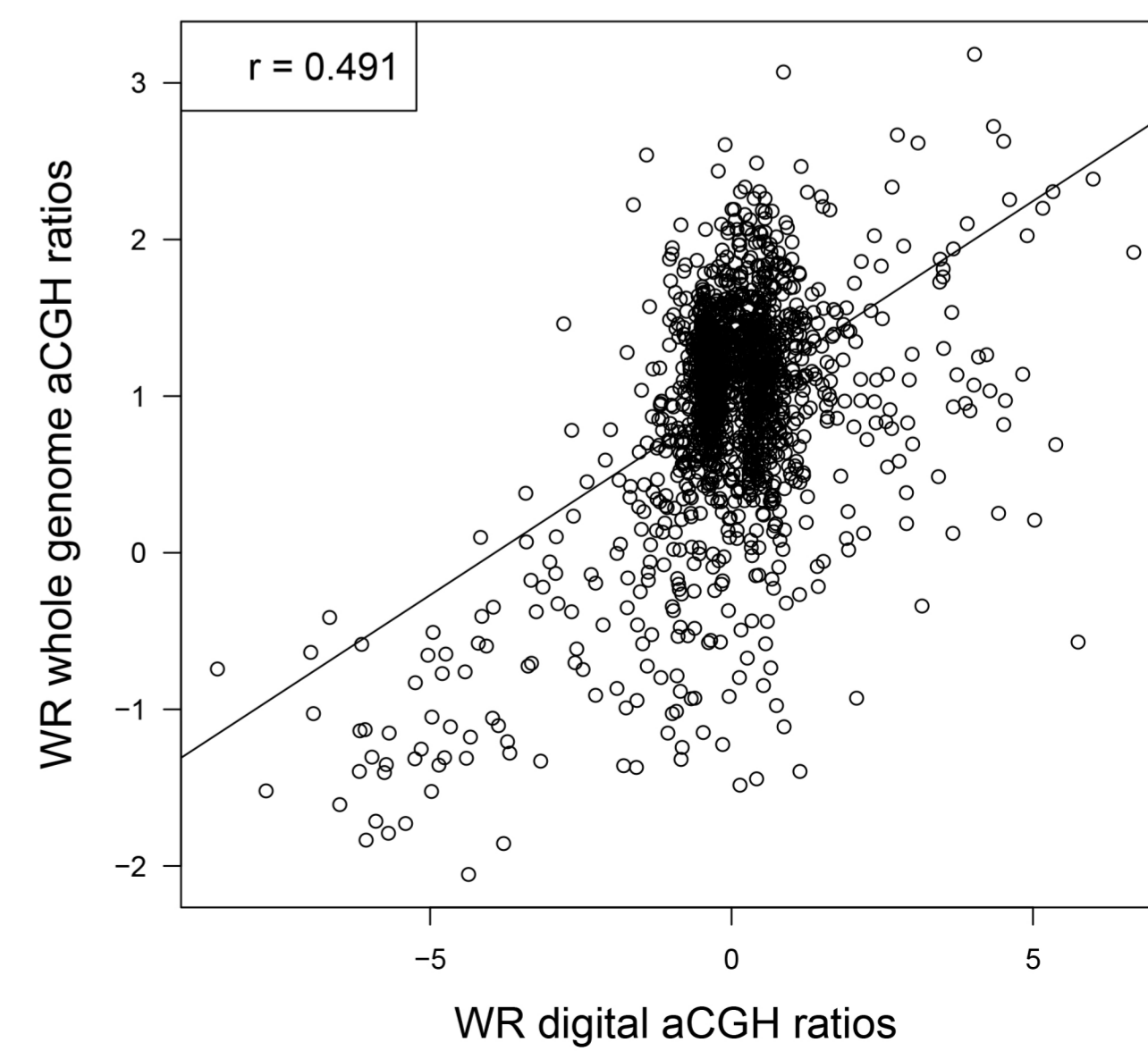

Supplement: Supplementary file 5 — Additional file 5: Figure S2: Correlation between digital aCGH and whole-genome aCGH among nine individuals compared with Red Jungle Fowl (RJF). RJF is selected as the reference sample in each aCGH experiment. Digital aCGH values are estimated using calculated log2 CN ratios in which CN are estimated for identified CNV segments of nine individuals and divided by the corresponding CN of RJF. Whole genome aCGH values are defined as the average of all probes log2 ratio values in the same segments as the digital aCGH. (PDF 7 MB) [file 12864_2014_6663_MOESM5_ESM.pdf]

A

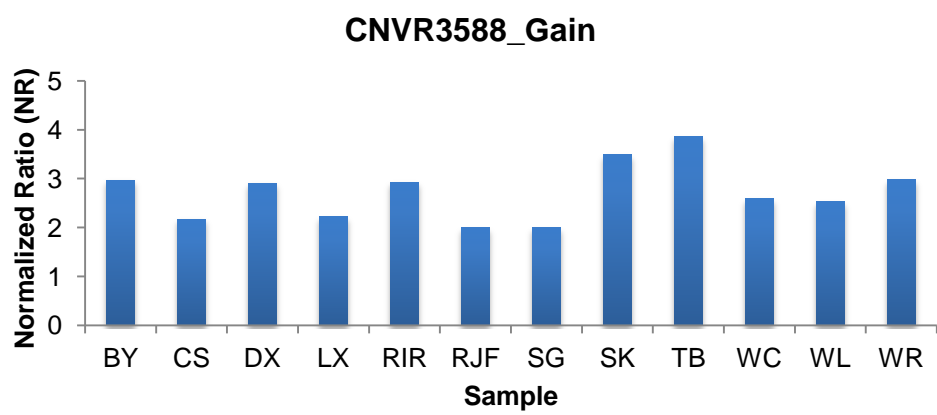

B

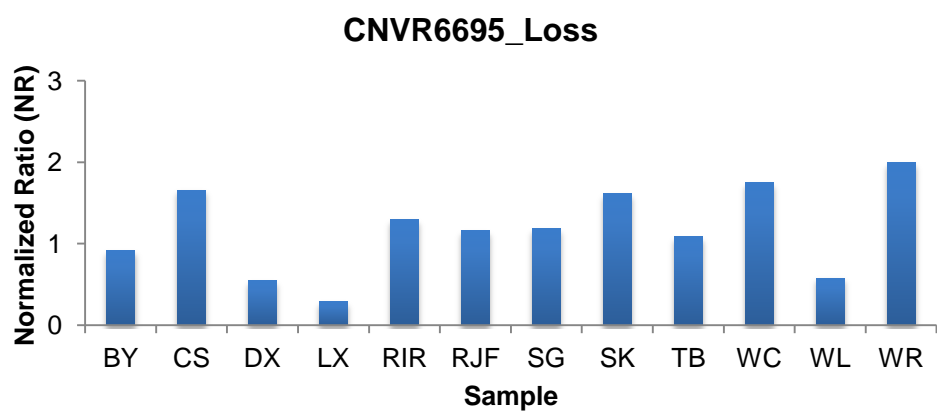

C

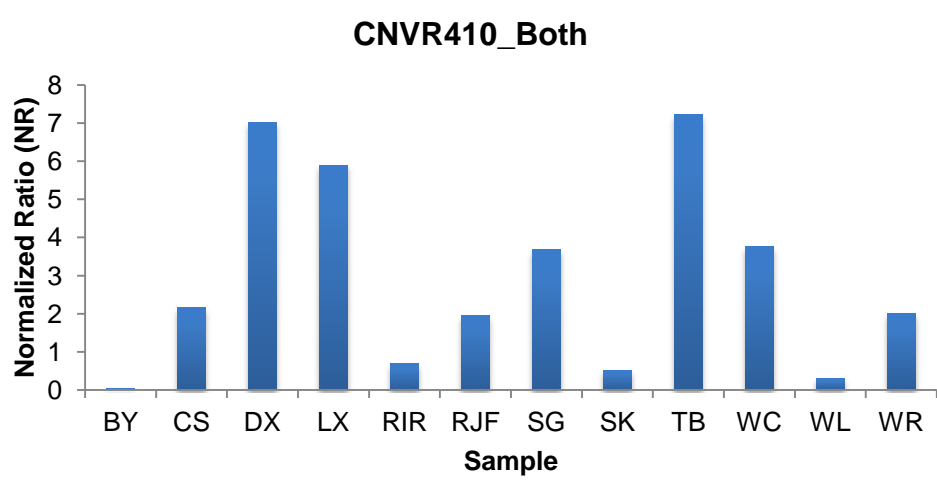

Supplement: Supplementary file 7 — Additional file 7: Figure S3: Illustrating of qPCR confirmation results for three selected CNVRs of different types. X-axis represents all 12 samples and Y-axis represents normalized ratios (NR) estimated by qPCR. NR around 2 indicates normal status (2 copies), NR around 0 or 1 indicates loss status (0 copies or 1 copy), and NR around 3 or more indicates gain status (3 or more copies). (A) Results for a gain status of CNVR3588. (B) Results for a loss status of CNVR6695. (C) Results for a both status of CNVR410. (PDF 63 KB) [file 12864_2014_6663_MOESM7_ESM.pdf]

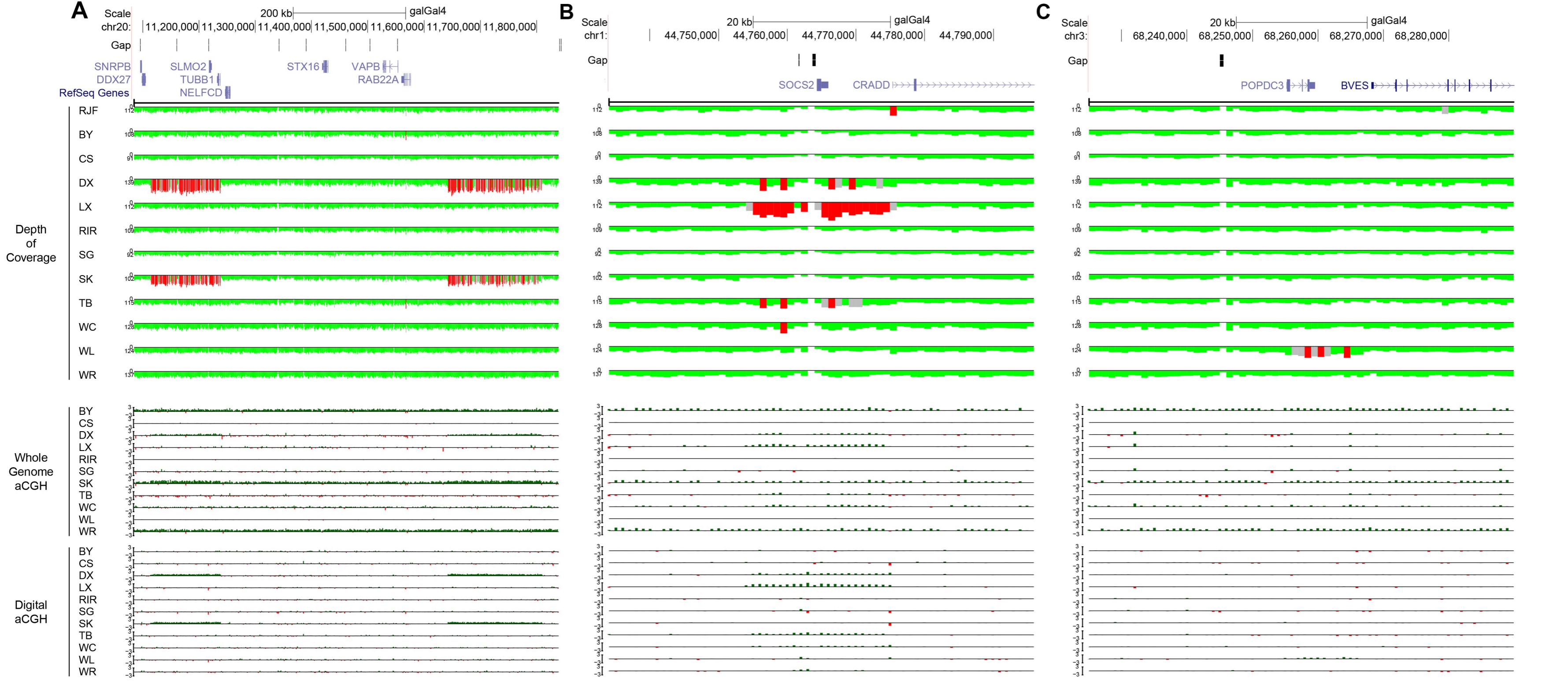

Supplement: Supplementary file 9 — Additional file 9: Figure S4: Visual examination by read depth, whole-genome aCGH and digital aCGH around three loci for 12 chicken genomes. The uppermost gene image is generated with the UCSC Genome Browser ( http://genome.ucsc.edu/) using the galGal4 assembly. The track below the gene region is depth of coverage for all 12 individual genomes. Red indicates regions of excess read depth (> mean +3 × STDEV), whereas gray indicates intermediate read depth (mean +2 × STDEV < × < mean +3 × STDEV), and green indicates normal read depth (mean ± 2 × STDEV). All read depth values based on 1 kb non-overlapping windows are corrected by GC content. Whole-genome aCGH and digital aCGH values are depicted as the red-green histograms and correspond to a gain colored in green (>0.5), a loss colored in red (<-0.5) and normal status colored in gray (-0.5 < x <0.5). (A) Two previously reported CNVs (chr20: 11,111,401-11,238,900 and chr20: 11,651,801-11,822,900) associated with dermal hyperpigmentation. The DX and SK genomes show two additional copies of the two regions compared with RJF, and are also validated by whole-genome aCGH. (B) A higher copy number increase for the SOCS2 locus (chr1: 44,764,280-44,765,955) is predicted in LX than in other individuals. (C) The POPDC3 gene (chr3: 68,255,196-68,259,535) is predicted to be duplicated status only in WL. (PDF 2 MB) [file 12864_2014_6663_MOESM9_ESM.pdf]
